# Shoot iron status and auxin are involved in iron deficiency-induced phytosiderophores release in wheat

**DOI:** 10.1186/s12870-018-1324-3

**Published:** 2018-06-04

**Authors:** Maria Garnica, Eva Bacaicoa, Veronica Mora, Sara San Francisco, Roberto Baigorri, Angel Mari Zamarreño, Jose Maria Garcia-Mina

**Affiliations:** 1 Department of Environmental Biology (BACh Group), School of Sciences, University of Navarra, Pamplona, Navarra Spain; 2 Plant Physiology and Plant-Microorganism Laboratory, University of Rio Cuarto, Río Cuarto, Cordoba Argentina; 3 Technical and Development Department, Timac Agro Spain, Lodosa, Navarra Spain

**Keywords:** Auxin, Hormones, Iron (Fe) deficiency, Nicotianiamine transferase, Phytosiderophores, Wheat

## Abstract

**Background:**

The release of phytosiderephores (PS) to the rhizosphere is the main root response to iron (Fe) deficiency in graminaceous plants. We have investigated the role of the Fe status in the shoot as well as of the signaling pathways controlled by three relevant phytoregulators – indolacetic acid (IAA), ethylene and nitric oxide (NO) – in the regulation of this root response in Fe-starved wheat plants. To this end, the PS accumulation in the nutrient solution and the root expression of the genes encoding the nicotianamine aminotransferase (*TaNAAT*) and ferritin (*TaFER*) have been evaluated in plants subjected to different treatments.

**Results:**

The application of Fe to leaves of Fe-deficient plants prevented the increase in both PS root release and *TaNAAT* gene expression thus showing the relevant role of the shoot to root communication in the regulation of PS root release and some steps of PS biosynthesis. Experiments with specific hormone inhibitors showed that while ethylene and NO did not positively regulate Fe-deficiency induced PS root release, auxin plays an essential role in the regulation of this process. Moreover, the application of IAA to Fe-sufficient plants promoted both PS root release and *TaNAAT* gene expression thus indicating that auxin might be involved in the shoot to root signaling network regulating Fe-deficiency root responses in wheat.

**Conclusions:**

These results therefore indicate that PS root release in Fe-deficient wheat plants is directly modulated by the shoot Fe status through signaling pathways involving, among other possible effectors, auxin.

## Background

The limitation of iron (Fe) bioavailability is one of the main problems affecting both the yield and quality of many crops cultivated in alkaline-calcareous soils [1, 2]. This fact is mainly related to the ability of Fe to form highly insoluble compounds in this type of soil, mainly hydroxides-oxides and carbonates [3]. In order to cope with this problem plants have developed specific coordinated mechanisms, both in roots and shoots, to optimize the functional use of Fe within the plant and Fe root uptake, as well as to increase the pool of potentially available Fe in the rhizosphere. However, these mechanisms are different depending on the plant species [4–8].

Under Fe deficiency non-graminaceous plants have evolved specific physiological responses in the root [4, 8, 9]. These responses, normally known as reduction-based strategy or Strategy I, have been extensively studied and characterized at both transcriptional and post-transcriptional levels. Strategy I responses include the activation of enzymatic Fe (III) reduction at the root surface (Fe (III) chelate reductase) and Fe (II) transport activity into the root (Fe (II)-transporter). The dominant genes responsible for these processes are ferric-chelate reductase oxidase gene FRO2 and the iron regulated transporter gene IRT1 [8]. The release of proton and phenolic compounds to the rhizosphere is another process included in Strategy I, inducing acidification by the activation of a root plasma membrane H^+^-ATPase coded by HA genes [4, 8, 9]. Recently genes involved in phenolic efflux transporters (PEZ) have been identified [8]. Induction of these molecular components is accompanied by morphological modifications in root architecture, such as an increase in secondary and lateral roots, absorbent hairs and transfer cells [10].

All these events in roots seem to be closely coordinated with other processes in shoots oriented to optimize the metabolic availability and use of Fe within the plant [11, 12]. In fact, even though isolated roots are able to activate root Fe-starvation main responses [13], it is the Fe-status in the shoot which modulates the activation of physiological Fe-starvation responses in roots [14–19].

On the other hand, graminaceous plants cope with Fe-deficiency by activating root responses different from those involved in Strategy I plants. These responses, known as chelation-based strategy or Strategy II responses, include the synthesis and release to the rhizosphere of specific compounds with iron chelating properties named phytosiderophores (PS) [4, 5, 8, 20, 21]. PS are organic compounds that belong to the family of mugineic acids synthesized from S-adenosyl-L-methionine (SAM). Sequential enzymatic reactions mediated by nicotianamine synthase (NAS), nicotianamine aminotransferase (NAAT) and deoxymugineic acid synthase (DMAS) generate deoxymugineic acid (DMA), the precursor of all types of mugineic acids (MAs) [22–24] Fe deficiency tolerance differs among species in graminaceous plants and is thought to depend mainly on the amount and types of MAs secreted [8]. The Fe-PS complex is then transported into root cells through a high affinity uptake root transporters, like TOM1 transporter of mugineic acid family phytosiderophores and YS1 and YSL1 of Fe(III)-MAs complexes transporters [8, 22]. As in the case of Strategy I responses, the gene network involved in the regulation of Strategy II responses have been well characterized at both molecular and physiological levels in some model plants, such as rice and barley [8, 25, 26]. Induction of Fe acquisition related genes help the plants cope with Fe shortage, as well as genetic regulatory factors such as DNA methylation and co-supression [26], and generating transgenic crops with improved nutritional traits [8]. For instance, rice secretes only small amounts of DMA among the MAs, and is more susceptible to low Fe availability compared with barley, a plant species with high capacity to produce MAs [8]. Introducing barley genome fragments containing Hv NAAT-A/B, HvNAS1 or IDS3 into rice resulted in increased DMA secretion and substantial tolerance of rice to calcareous soils under paddy conditions [27]. However, the role of the shoot Fe status in the systemic regulation of Fe-deficiency root responses involved in their regulation remains unclear.

Several common signaling molecules affect the Fe deficiency response, like plant hormones. Moreover, a number of studies have shown the crucial role of a coordinated action of IAA, ethylene and NO in the signaling network involved in both shoot to root communication and Fe-deficiency root responses activation [17, 18, 28–31].

Some studies reported that NO may affect Fe-deficiency root responses in graminaceous plants by improving internal Fe availability and further use efficiency [32]. NO production contributes to the improvement of Fe acquisition and homeostasis by regulating the expression of Fe-related genes under Fe deficiency, such as modulating ferritin genes and its accumulation in leaves [33]. Furthermore, NO modulated the physiological and morphological responses to Fe shortage [34], like regulating lateral root formation in Fe deficient plants [35].

With regard to ethylene (ETH) Welch et al. [36] reported that the application of the ETH precursor 1-aminocyclopropane-1 carboxylic acid (ACC) to barley plants did not affect PS root release under Fe-starvation. However, the application of amino-oxiacetic acid (AOA), an ethylene inhibitor, increased PS root release but negatively affected both Fe root uptake and whole plant growth [34]. Romera et al. [37] did not observe any increase of ethylene root production associated with Fe-deficiency in several graminaceous species. However, Wu et al. [38] observed that Fe deficiency increases ethylene root production in rice. These results are only apparently contradictory because rice seems to have both strategy I and strategy II root responses to Fe deficiency [39–41]; as is also the case with maize [42].

As for a potential role of auxin-dependent signaling pathways, Qi et al. [43] described that *Os*ARF12, a transcription factor that activates the auxin response gene, affected Fe accumulation and distribution in rice. Furthermore, Xu et al. [44] also described a new gene, *Os*ABCB14, involved in both auxin transport and Fe homeostasis in rice. Recently, Liu et al. [41] reported that auxin signaling is involved in the regulation of Fe-deficiency induced impairment of photosynthetic activity and shoot growth in rice, as well as in the activation of the main gene codifying the Strategy I main responses in shoots. These results are in line with previous studies showing the relevant role of auxin in the regulation of Strategy I responses under Fe deficiency [28, 29]. Bacaicoa et al. [30] reported that IAA is involved in the regulation at the transcriptional and functional levels of both Fe root acquisition (Fe chelate reductase, Fe transport) and rhizosphere adcidification (H+ ATPase) in cucumber plants, although through different and complementary mechanisms. However, the results concerning genes involved in Strategy II responses (*Os*YSL15 and *Os*DMAS1) were less conclusive [41]. Recently, Kabir et al. [45] reported that a shoot-based auxin signaling is involved in the resistance of wheat to Fe toxicity. In summary, there exists experimental evidence suggesting that auxin is involved in Fe homeostasis in graminaceous plants but its specific role remains unclear.

With regard to the potential role of other plant hormones, a recent study reporting the transcriptomic profiles of maize roots in response to Fe deficiency showed that genes involved in signaling pathways controlled by gibberellin, cytokinin and brassinosteroids (BRS) were significantly up-regulated. These results are in line with those reported by Wang et al. [46, 47] showing that BRS probably play a role in iron homeostasis in both Strategy I (cucumber) and Strategy II (rice) plants. Likewise, a recent study showed that genes also regulated by jasmonate were also upregulated in rice plants subjected to Fe deficiency [48].

In this framework, it is possible that Fe-deficient root responses in graminaceous plants are also modulated by the shoot Fe status through signaling pathways involving one or several phytoregulators. In order to investigate this hypothesis, we have studied whether the Fe-status in the shoot modulates PS secretion in roots, as well as the role of IAA, NO and ethylene in the regulation of this process, in wheat plants subjected to Fe deficiency. With this aim, we have used a pharmacological approach involving inhibitors of the biosynthesis and/or action of IAA, ethylene and NO. Likewise, the role played by the shoot Fe-status in PS root release was also assessed through foliar FeSO_4_ resupply to Fe-starved wheat plants. The PS-related root responses under Fe-deficiency were studied using three approaches:

(i) The study of the expression of the gene encoding nicotianamine aminotransferase (TaNAAT) in roots. This gene plays a key role in the biosynthesis pathway of PSs (PSs-mugeneic family) from nicotianamine.
(ii) The study of the expression of the gene encoding ferritin biosynthesis in leaves (*TaFer1* and *TaFer2*), which plays a key role in Fe internal bioavailability in leaves [8, 49].
(iii) The study of the concentration of PSs in the nutrient solution.

## Methods

### Plant culture and experimental design

Seeds of *Triticum aestivum* cv. *Bermude* were germinated with distilled water in 300 ml plastic opaque pots containing perlite, in a germinating chamber in the darkness and at a temperature of 25 °C and 85% of relative humidity, for 10 days. After germination, the seedlings were grouped into two groups, one receiving Fe and the other without Fe, and transferred to 8 L pots and grown in aerated hydroponic culture for 11 days in a growth chamber. Fe-sufficient plants were grown with 89 μM EDTA-Fe, while Fe-deficient plants were grown without Fe in order to induce iron deficiency. The nutrient solution containing the other nutrients were the same for all plants and contained 5 mM N, 5 mM K, 4 mM Ca, 1 mM P, 1 mM S, 1 mM Mg, 18 μM Mn, 0.9 μM Cu, 1.75 μM Zn. The nutrient solutions were renewed every week. The initial pH of the nutrient solution was 6.0 and did not show significant changes during the experiment. The growth chamber conditions were as follow, temperature of 24/18 °C and 50–70% relative humidity with a 15/9 h day/night photoperiod (irradiance: 250 μmol m^− 2^ s^− 1^ photosynthetically active radiation).

After 11 days, the plants belonging to the two groups (+Fe and –Fe) were transferred to renewed nutrient solutions and the different pharmacological treatments were applied: IAA- or ethylene- inhibitors (for Fe-starved plants), a NO-scavenger (for Fe-starved plants), or IAA (for Fe-sufficient plants) depending on the experiment carried out. Final doses of the different inhibitors used, as well as IAA doses, were selected according to previous results obtained from time-course, dose-response preliminary experiments (data not shown). Each treatment consisted of three replicates with 20 plants per replicate.

In experiments focused on the study of the functional role of IAA on root responses to Fe deficiency, the following IAA inhibitors and doses were used: 2-(p-chlorophenoxy)-2-methylpropionic acid (PCIB, 200 μM) and 2,3,5-triiodobenzoic acid (TIBA, 50 μM). PCIB was used dissolved in dimethyl sulfoxide (DMSO) and added to the nutrient solution (the same volume of DMSO was added to control treatments). TIBA was used dissolved in methanol and added to the nutrient solution (the same volume of methanol was added to the control treatments). In experiments focused on the ability of IAA to activate PS root release in plants growing in Fe-sufficient conditions, doses of 0.1 μM and 10 μM of IAA were applied to the nutrient solution of Fe-sufficient plants.

In order to investigate the role of ethylene and NO in the study of Fe-deficiency root responses, silver thiosulphate (STS, 100 μM) and cobalt as cobalt nitrate (Co^2+^, 5 μM) were used as ethylene inhibitors. As an NO-scavenger, 2-phenyl- 4,4,5,5-tetramethylimidazoline-3-oxide-1-oxyl (c-PTIO, 50 μM) was used. Both were supplied in the nutrient solution.

In experiments focused on the potential role of shoot Fe status in the regulation of the activation of Fe-deficiency root responses, 3.6 mM Fe as FeSO_4_ was applied on the leaves of Fe-starved plants. Foliar treatments contained 0.1% of a surfactant, in order to ensure a good distribution of sprayed solution on the leaf surface (Biopower; sodic alkyl ether sulfate; Bayer Crop-Science). Previous studies showed that this surfactant, used at 0.1%, did not affect Fe-root stress responses at both transcriptional and post-transcriptional levels (data not shown).

Harvests were carried out at 4, 72 and 96 h upon pharmacological treatments application. The harvests were conducted at the same time of the day to exclude diurnal variations, which meant 8 h after the start of the light period. Shoots and roots were dried at 60 °C for dry matter evaluation and the analysis of total Fe and the Fe fraction that is soluble in 0.1 M HCl. The total Fe was determined after acidic digestion of dried samples, and soluble Fe was determined after extraction of fresh samples in 0.1 M HCl (1:10) for 16 h at room temperature. Analyses were carried out by ICP-OES. A specific portion of the shoots and roots was quick-frozen in liquid nitrogen for hormone and gene expression analyses.

Root exudates were collected for the measurement of phytosiderophore release from roots at each harvest time. For the collection of root exudates, intact plants were removed from the nutrient solution and the roots were washed with deionized water. Thereafter, plants were placed in 200 ml aerated distilled water for 3 h. Root exudates were collected and frozen at − 40 °C following previous treatment with Micropur (Katadyn, Switzerland) to prevent microbial degradation.

### Phytosiderophores determination

Phytosiderophores were measured as described in Reichman and Parker [50]. Briefly, a 10 ml aliquot of sample solution was dispensed into a vial and 10 ml of a blank of deionized water was dispensed into a separate vial. 0.5 ml of 0.6 mM FeCl_3_ was added to each vial. All vials were shaken for 15 min, and then 1 ml of 1.0 M Na-acetate buffer (pH 7.0) was added and the solutions were shaken for 10 min. To reduce Fe (III) to Fe (II), the solutions were filtered through a 0.2 μm filter into 0.25 ml of 6 M HCl and then 0.5 ml of 80 g L^− 1^ hydroxylamine hydrochloride was added. All the solutions were placed in an oven at 50–60 °C for 30 min. After incubation 0.25 ml of 2.5 g L^− 1^ Ferrozine and 1 ml of 2.0 M Na-acetate buffer (pH 4.7) were added to the solution. Finally, the tubes were shaken briefly to mix the contents and after 5 min the absorbance at 562 nm was determined. The absorbance readings were converted into concentration using the Beer-Lambert law against a reference curve prepared with adequate Fe standards. It was assumed that the stoiquiometry of reaction is 1:1.

### Extraction and quantification of IAA in plants tissues

The extraction and purification of IAA, as well as its quantification, was carried out according to the method described in Bacaicoa et al. [30].

### Real-time quantitative RT- PCR analysis

The roots of the plants were collected and ground to a powder with liquid nitrogen prior to RNA extraction. Total RNA was extracted from between 50 and 90 mg of crushed root using a mix of 350 μL of guanidinium-thiocyanate lysis buffer and 3.5 μL of β-mercaptoethanol of NucleoSpin RNA Plant Kit (Macherey-Nagel, Düren, Germany). Following this, treatment of RNA with DNase was performed according to the manufacturer’s recommendations. After washing extracted RNA with dry silica membranes provided by the kit, RNA purity and concentration was quantified by fluorescence-based Experion RNA STdSens Analysis kit. First-strand cDNA synthesis was carried out in 20- μL reactions containing 1 μg of RNA with RNase H+ MMLV reverse transcriptase iScript and a mix of oligo(dT) and random hexamer primers from iScript cDNA Synthesis Kit (Bio-Rad Laboratories, Hercules, CA). The reverse transcription was made up for 5 min at 25 °C, 30 min at 42 °C, and ended by 5 min at 85 °C. The gene expression was analyzed with the CFX384 Touch Real-Time qPCR detection System (Bio-Rad Laboratories) using iQ SYBR Green supermix (Bio-Rad) containing hot-start iTaq DNA polymerase in 10- μL reaction volume with 1 μL of cDNA.

Primer pairs used to amplify wheat nicotianamine transferase *Ta*NAAT were designed from BT009504. Sense primer5´- TCATCATAAACCCAAACAATCC-3′ and antisense 5′- TATACCTCGTCAGCAATCAC-3′.

Primer pairs used to amplify wheat ferritin genes were those described in Borg et al. (2012)51. *Ta*Fer1: sense 5’-GGCTCCAGTCAATCGTCACA-3′ and antisense 5’-ACAAAGTCGGTCAGCTGAGG-3’.

Primers were synthesized by Sigma-Genosys (Cambridge, United Kingdom). Standardization was carried out based on the expression of the *Triticum aestivum* RNase L-inhibitor-like protein (RLIa) gene in each sample, using corresponding specific primers (Unigene Ta2776). Data analysis of the relative abundance of the transcripts was done using CFX manager Software Data Analysis (Bio-Rad Laboratories). Data were normalized with respect to the transcript level of the reference gene with the normalized expression method (∆∆Ct). Expression analyses were carried out on three independent root RNA samples and repeated three times for each RNA sample.

### Statistical analysis

Significant differences (*p* ≤ 0.05) among treatments were calculated by using one-way analysis of variance (ANOVA) and the HSD Tukey post hoc test. All statistical tests were performed using the statistical package Statistica 6.0 (StatSoft, Tulsa USA).

## Results

### Plant model validation

**Fig. 1:**
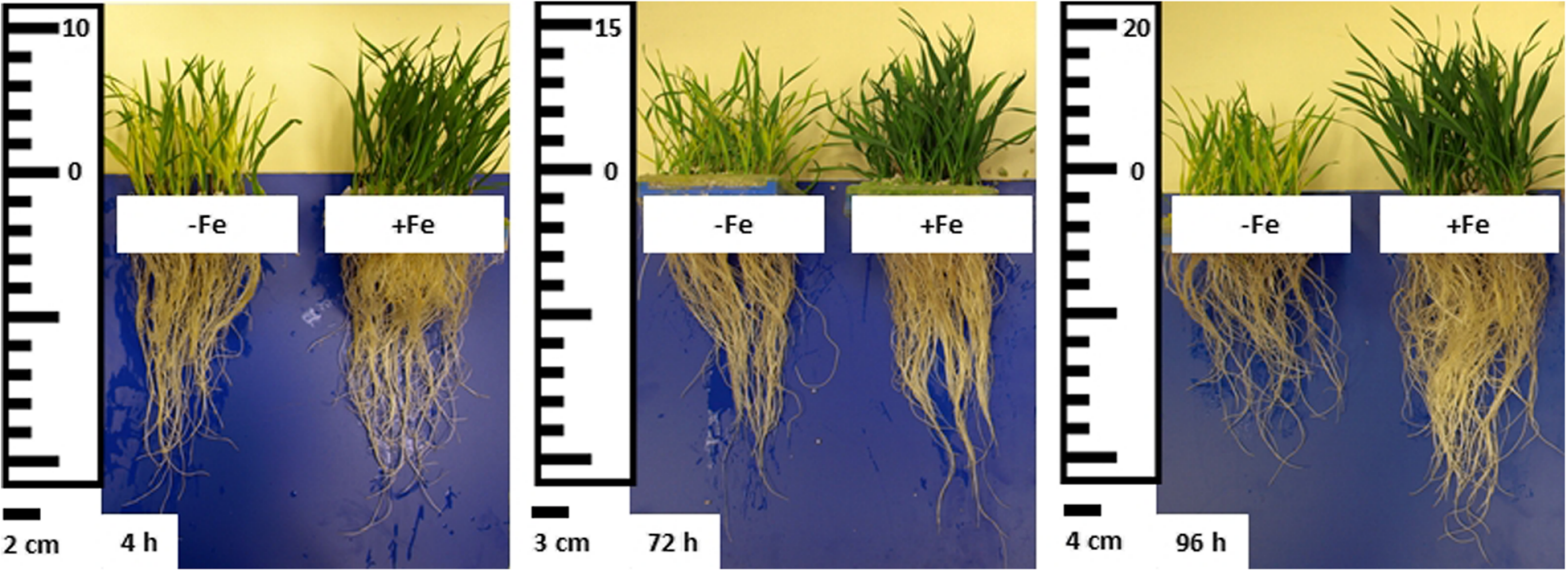
Morphological effects on wheat Fe-sufficient plants (+Fe) and wheat Fe-starved plants (-Fe)

An initial experiment was carried out in order to validate our plant model for Fe deficiency studies in wheat. Fe-starved plants presented a clear reduction in the growth of both shoot and root, as well as an intense chlorosis in leaves that was reflected in the decrease in leaf chlorophyll measured in SPAD units (Table 1, Fig. 1). Likewise, Fe- deficiency was accompanied by a significant decrease in both total and soluble Fe, as well as the release of PS from the root to the nutrient solution.

**Table 1:** Physiological parameters under Fe sufficient and Fe starved treatments

Shoot 4 h	DW	Total Fe	Sol. Fe	SPAD	PN	PS	IAA
-Fe	1.38 (0.05)	36.6 (2.38)	29.4 (3.65)	28.8 (1.53)	3.95 (0.97)	–	31.1 (4.70)
+Fe	1.68 (0.17)	76.0* (5.62)	57.5* (2.51)	39.1* (1.31**)**	4.82 (0.57)	–	34.6 (5.47)
Root 4 h							
-Fe	0.90 (0.19)	45.0 (2.29)	–	–	–	4.98* (1.91)	20.6 (1.47)
+Fe	0.81 (0.08)	640* (25.4)	–	–	–	1.36 (0.47)	39.2* (7.52)
Shoot 72 h	DW	Total Fe	Sol. Fe	SPAD	PN	PS	IAA
-Fe	1.72 (0.18)	39.6 (1.25)	29.8 (1.48)	19.7 (1.65)	2.65 (0.56)	–	29.5 (4.89)
+Fe	2.90* (0.25)	123* (27.8)	92.6 (3.75)	39.4* (2.50)	4.08* (0.49)	–	29.8 (2.50)
Root 72 h							
-Fe	1.08 (0.18)	39.0 (4.75)	–	–	–	2.63* (0.96)	25.8 (5.15)
+Fe	1.13 (0.25)	294* (10.8)	–	–	–	0.18 (0.04)	41.9* (3.36)
Shoot 96 h	DW	Total Fe	Sol. Fe	SPAD	PN	PS	IAA
-Fe	1.74 (0.20)	40.9 (5.15)	22.2 (2.02)	22.7 (0.64)	2.28 (0.64)	–	31.9 (6.52)
+Fe	2.94* (0.07)	88.6* (6.05)	67.7* (5.42)	43.8* (2.41)	4.10* (0.35)	–	31.7 (6.80)
Root 96 h							
-Fe	1.10 (0.16)	73.8 (9.04)	–	–	–	5.64* (1.36)	28.6 (6.45)
+Fe	1.34 (0.16)	495* (6.11)	–	–	–	0.60 (0.36)	44.2* (1.29)

Dried Weight: DW (g), Total Iron: Total Fe (μg·g^−1^), Soluble Iron: Sol. Fe (μg·g^− 1^), Chlorophyll content as SPAD measure in arbitrary units SPAD (a.u.), Photosynthetic Rate: PN (μmol·m^− 2^·s^− 1^), Phytosiderophore concentration: PS (μmol·g^− 1^ FW), Indole-acetic acid: IAA (pmol·g^− 1^ FW). Standard deviation in parentheses. *means statistical differences between treatments, *p* < 0.05 (Tukey HSD test)

### Foliar Fe supply to Fe-deficient plants avoided root PSs release and *TaNAAT* gene up-regulation

**Fig. 2:**
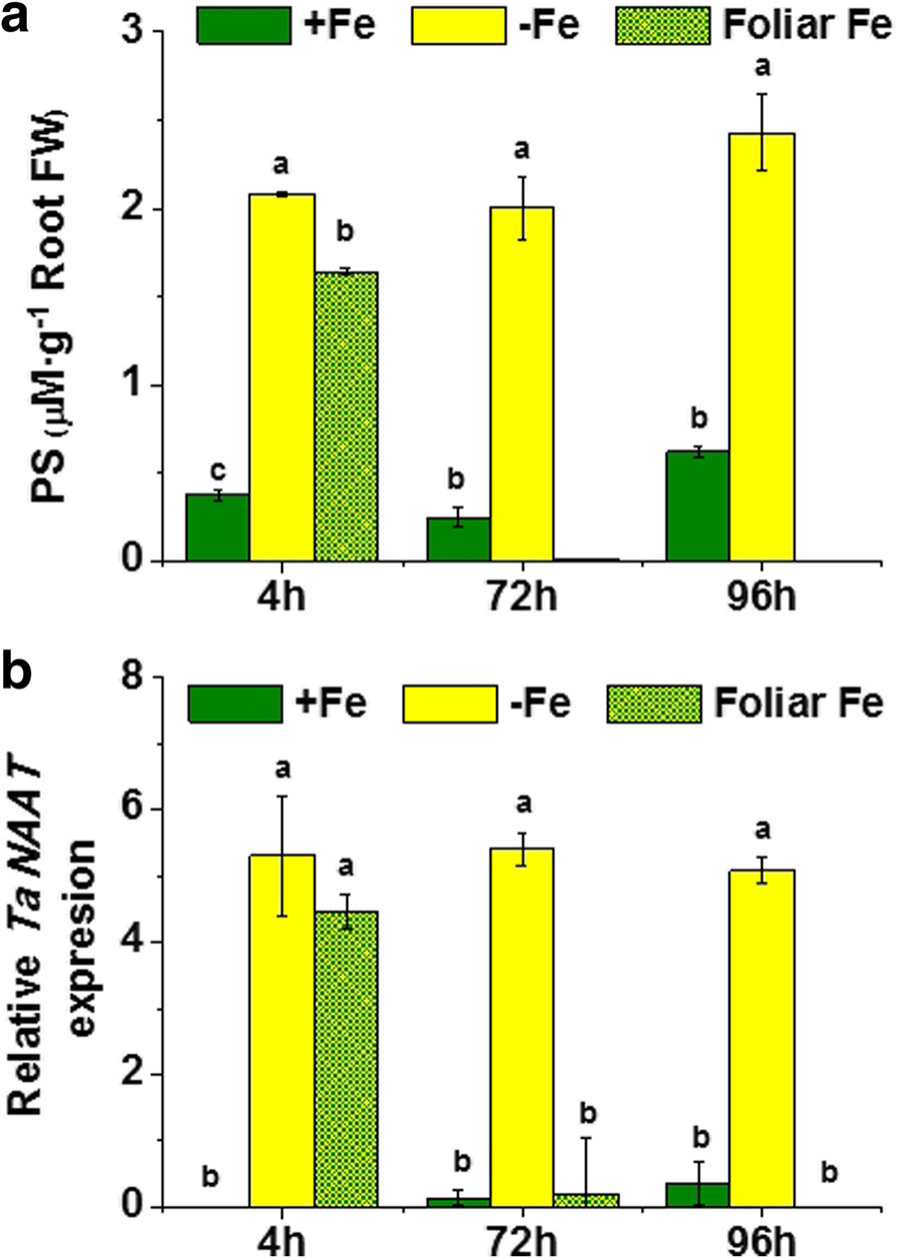
Effect of iron resupply on wheat plants under iron deficiency. **a** Phytosiderophores (PS) release by Fe-sufficient plants (+Fe), Fe-starved plants (-Fe) and Fe supplied to Fe-starved plants (Foliar Fe) in μM (assuming a Fe-PS chelation 1:1) per g of root fresh weight. Average of 3 replicates with 20 plants each. *p* < 0.05 (Fisher LSD test). **b** Relative root *Ta*NAAT gene expression in Fe-sufficient plants (+Fe), Fe-starved plants (-Fe) and Fe supplied to Fe-starved plants (Foliar Fe). Average of 3 replicates with 20 plants each. *p* < 0.05 (Tukey HSD test)

Fe starvation caused a significant increase in both PS root release and *TaNAAT* gene expression in roots (Fig. 2a, b). In order to investigate whether the Fe-status in the shoot plays a dominant role in the regulation of these root responses to Fe-deficiency, we studied the effect of the application on the leaves of Fe-deficient plants of foliar sprays containing water-soluble Fe, on both root *TaNAAT* gene expression and the PS release to the nutrient solution.

The results showed that both *TaNAAT* gene expression in roots and PS root release were reduced 4 h upon Fe-foliar treatment compared to control Fe-deficient plants and disappeared after 72 and 96 h (Fig. 2a, b).

These results indicate that the Fe-status in the shoot modulates the activation of Fe-deficiency root responses in wheat.

### Ethylene inhibitors did not avoid PS root release and *TaNAAT* gene up-regulation in Fe-deficient plants

**Fig. 3:**
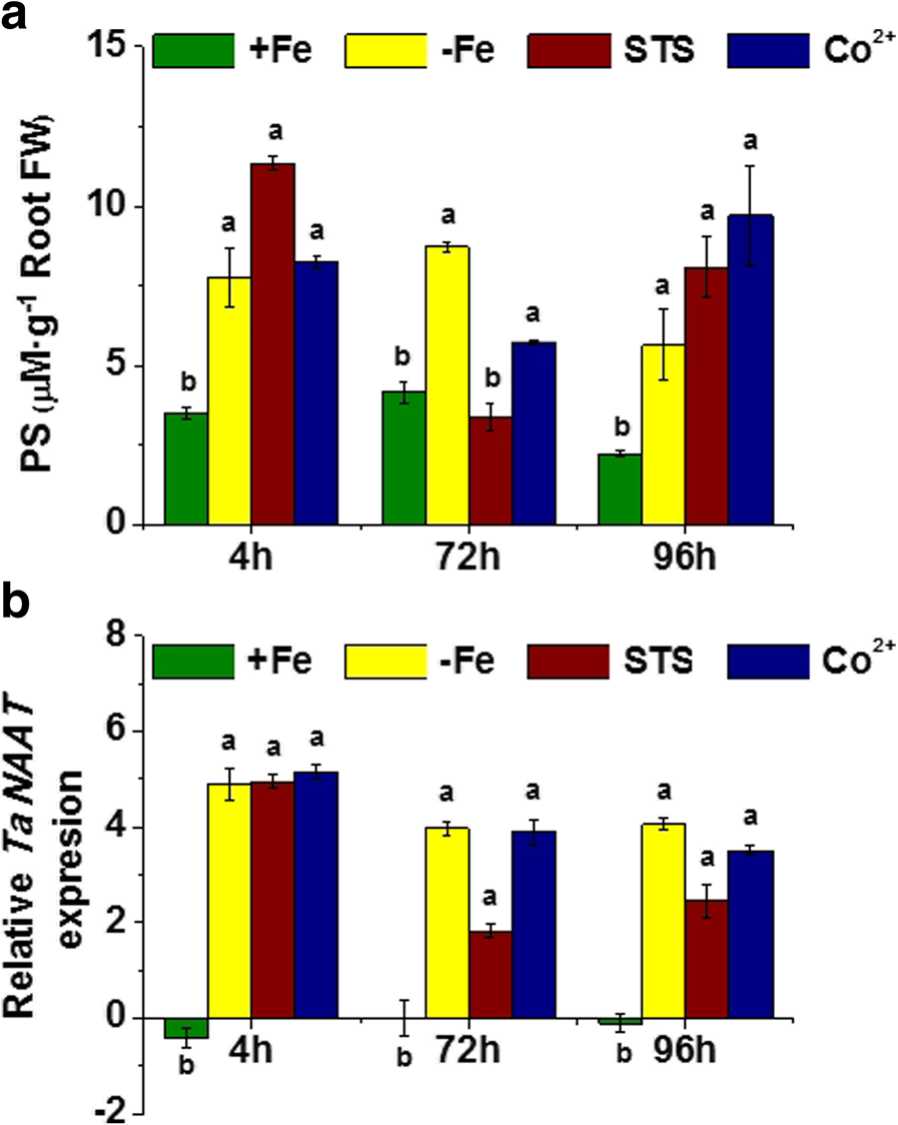
Effect of ethylene inhibitors supply on wheat plants under iron deficiency. Harvest were performed at 4, 72 and 96 h of treatment, which corresponded to the three columns per treatment (**a**) Phytosiderophores (PS) release by Fe sufficient plants (+Fe), Fe-starved plants (-Fe), and Fe-starved plants plus ethylene inhibitors: STS (100 μM), Co^2+^ (5 μM) plants in μM (assuming a Fe-PS chelation 1:1) per g of root fresh weight. Average of 3 replicates with 20 plants each. *p <* 0.05 (Fisher LSD test). **b** Relative root *Ta*NAAT gene expression in Fe sufficient plants (+Fe), Fe-starved plants (-Fe), and Fe-starved plants plus ethylene inhibitors: STS (100 μM), Co^2+^ (5 μM) plants. Average of 3 replicates with 20 plants each. *p* < 0.05 (Tukey HSD test)

Previous studies indicated that ethylene affected plant root responses to Fe starvation in graminaceous plants [38]. In order to assess whether ethylene positively regulates the main Fe-deficient root responses, we investigated the effect of two different ethylene inhibitors - silver thiosulfate (STS), an inhibitor of ethylene action; and Co^2+^, an inhibitor of ethylene biosynthesis, on PS root release and root *TaNAAT* expression, in Fe-deficient wheat plants. The results obtained showed that the presence of either Co^2+^ or STS did not consistently affect PS release to the nutrient solution in Fe-deficient plants (Fig. 3a).

On the other hand, STS significantly reduced *TaNAAT* expression with respect to Fe-starved plants for 72 and 96 h upon treatments, although *TaNAAT* expression levels were significantly higher than those for Fe-sufficient plants (Fig. 3b). Co^2+^ did not affect *TaNAAT* expression in Fe-deficient plants (Fig. 3b).

These results indicate that ethylene does not positively modulate the studied Fe-deficiency root responses in wheat.

### Application of c-PTIO did not affect the studied Fe-deficiency root responses

Previous studies reported the ability of NO to enhance Fe availability within the plant in both graminaceous and dicotyledonous plants subjected to Fe starvation, thus mitigating the deleterious effects of Fe deficiency [32]. It is likely that this action of NO might modulate in some way root plant responses to Fe deficiency. In addition, some studies showed that NO works as a secondary messenger of auxin signaling in Strategy I root responses to Fe deficiency [28]. In order to investigate this hypothesis, we studied the effect of the application of a NO-scavenger (c-PTIO) to the nutrient solution of Fe-deficient plants on the expression of genes encoding *TaNAAT* in roots and ferritin in leaves (*TaFer1*), as well as PS root release.

**Fig. 4:**
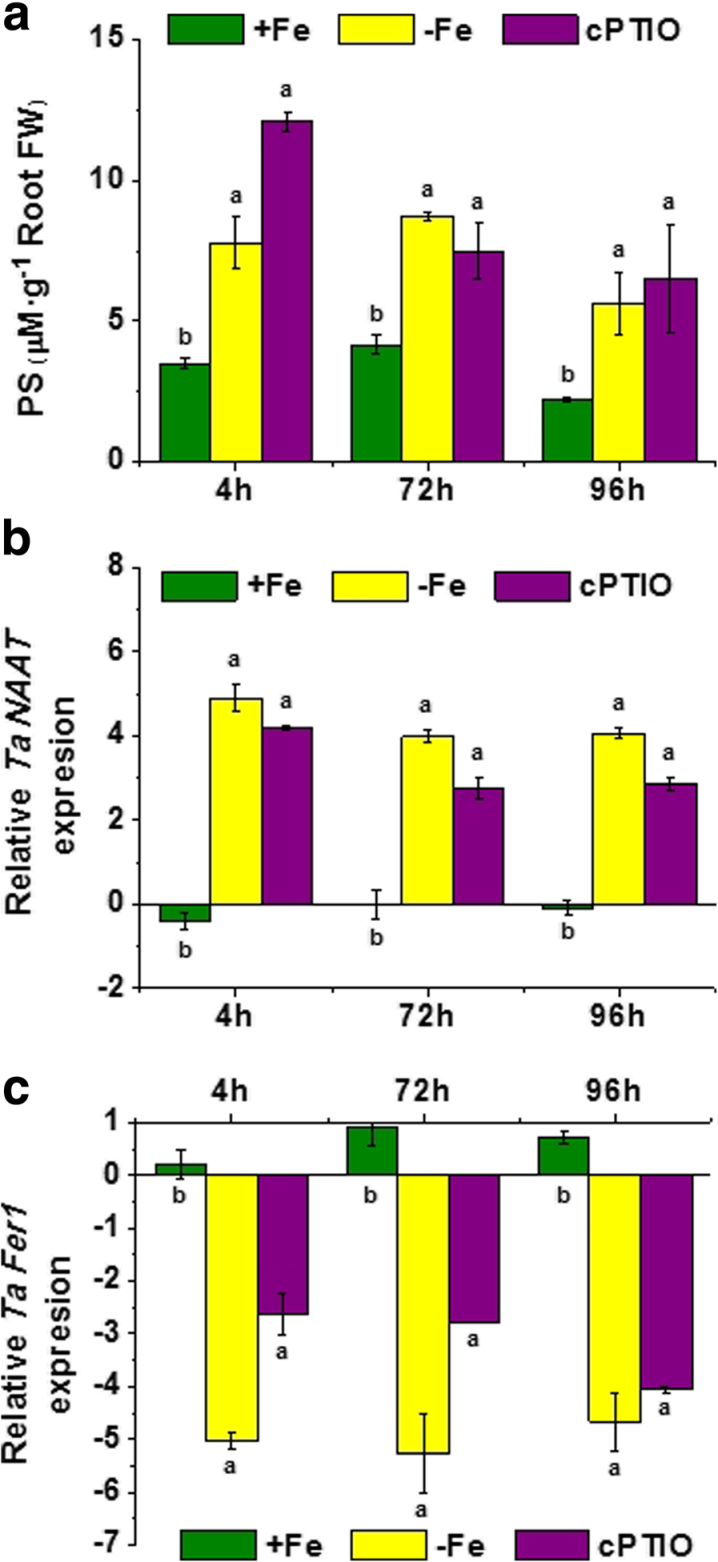
Effect of nitric oxide inhibitor supply on wheat plants under iron deficiency. Harvests were performed at 4, 72 and 96 h of treatment, which corresponded to the three columns per treatment (**a**) Phytosiderophores (PS) release by Fe-sufficient (+Fe), Fe-starved (-Fe), and Fe-starved plants treated with the NO-scavenger c-PTIO (50 μM); in μM (assuming a Fe-PS chelation 1:1) per g of root fresh weight. Average of 3 replicates with 20 plants each. *p <* 0.05 (Fisher LSD test). **b** Relative root *Ta*NAAT gene expression in Fe-sufficient (+Fe), Fe-starved (-Fe), and Fe-starved plants treated with the NO-scavenger c-PTIO (50 μM). Average of 3 replicates with 20 plants each. *p <* 0.05 (Fisher LSD test). (**c**) Relative root TaFer1 gene expression in Fe-sufficient (+Fe), Fe-starved (-Fe), and Fe-starved plants treated with the NO-scavenger c-PTIO (50 μM). Average of 3 replicates with 20 plants each. *p* < 0.05 (Tukey HSD test)

The results showed that c-PTIO application to Fe-deficient plants did not affect PS release to the nutrient solution, as well as *TaNAAT* up-regulation in roots (Fig. 4a, b). On the other hand, results showed that Fe-deficiency caused a significant down-regulation of *TaFer1*, a fact that was not affected by c-PTIO application (Fig. 4c).

### Inhibition of IAA transport and functionality blocked both the PS root release and the root *TaNAAT* up-regulation in Fe-deficient wheat plants.

A number of studies suggested that auxin-signaling pathways seem to be involved in the plant ability to grow under Fe deficiency in graminaceous plants [41, 43, 44]. It is thus possible that, as in the case of dicotyledonous plants, auxin plays relevant roles in Fe-deficiency root responses also in graminaceous plants. In order to challenge this hypothesis, we investigated the effect of the application of two IAA inhibitors - an inhibitor of IAA transport within the plant (TIBA) and an inhibitor of IAA action (PCIB) – on both root *TaNAAT* gene expression and PS root release, in Fe-deficient wheat plants.

**Fig. 5:**
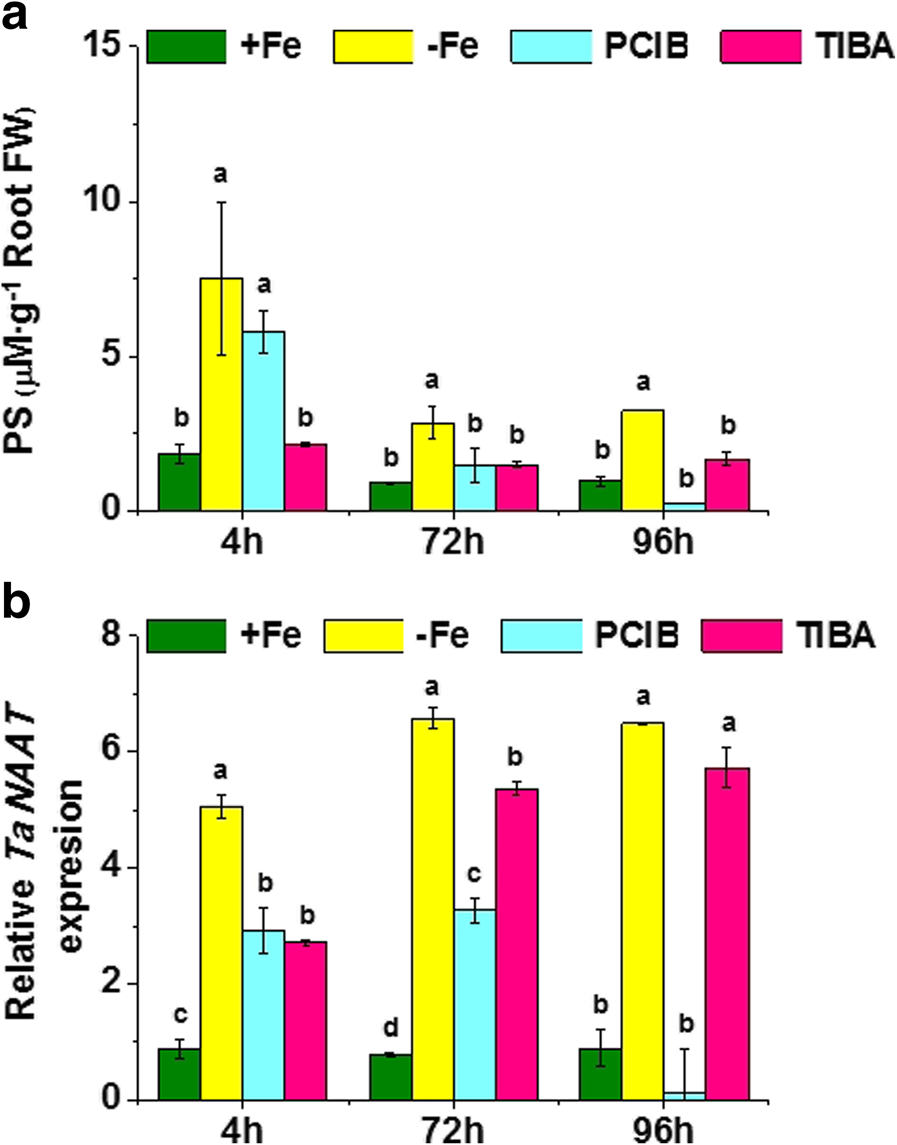
Effect of indole acetic acid inhibitors supply on wheat plants under iron deficiency (**a**) Phytosiderophores (PS) release by Fe-sufficient (+Fe), Fe-starved (-Fe), and Fe-starved plants treated with PCIB (200 μM) or TIBA (50 μM); in μM (assuming a Fe-PS chelation 1:1) per g of root fresh weight. Harvests were performed at 4, 72 and 96 h of treatment, which corresponded to the three columns per treatment. Average of 3 replicates with 20 plants each. *p <* 0.05 (Fisher LSD test). **b** Relative root TaNAAT gene expression in Fe-sufficient (+Fe), Fe-starved (-Fe), and Fe-starved plants treated with PCIB (200 μM) or TIBA (50 μM). Harvests were performed at 4, 72 and 96 h of treatment, which corresponded to the three columns per treatment. Average of 3 replicates with 20 plants each. *p* < 0.05 (Tukey HSD test)

The results obtained showed that the inhibition of IAA transport by the application of TIBA significantly reduced *TaNAAT* gene expression in Fe-deficient plants only 4 h after its application, while it reduced PS root release for all harvest times (Fig. 5a, b). However, PCIB application blocked *TaNAAT* gene up-regulation in roots for all harvest times and PS root release after 72 and 96 h, in Fe-deficient wheat plants (Fig. 5a, b). These results indicate that auxin-signaling pathways play a crucial role in the activation of studied root responses to Fe-deficiency in wheat, with this action being related to auxin transport and distribution.

### IAA root application induced root *TaNAAT* gene expression and PS root release in Fe-sufficient wheat plants

In order to investigate whether IAA is involved in the role of shoot Fe-status in the positive modulation of Fe-deficient root responses, we studied the effect of IAA application to roots of Fe-sufficient wheat plants on both root *TaNAAT* gene expression and PS root release. If IAA participates in the activation signaling network, its application to Fe-sufficient plants must activate to some extent, at least, Fe-deficiency root responses.

**Fig. 6:**
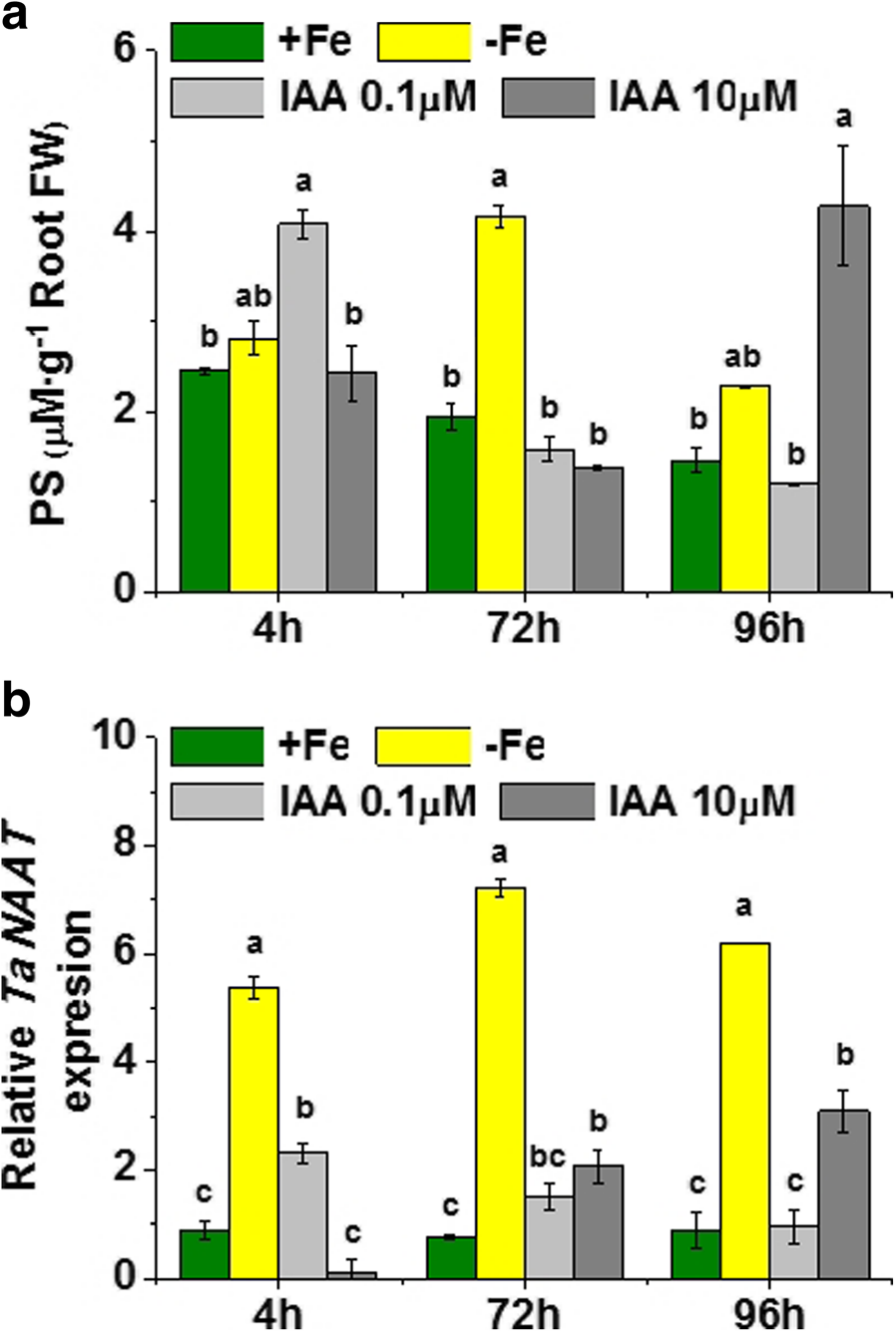
Effect of indole acetic acid (IAA) supply on Fe-sufficient wheat plants (**a**) Phytosiderophores (PS) release by Fe-sufficient plants (+Fe) treated with IAA (0.1 or 1 μM); in μM (assuming a Fe-PS chelation 1:1) per g of root fresh weight. Harvests were performed at 4, 72 and 96 h of treatment, which corresponded to the three columns per treatment. Average of 3 replicates with 20 plants each. *p* < 0.05 (Tukey HSD test). **b** Relative root *TaNAAT* gene expression in Fe-sufficient plants (+Fe) treated with IAA (0.1 or 1 μM). Harvests were performed at 4, 72 and 96 h of treatment, which corresponded to the three columns per treatment. Average of 3 replicates with 20 plants each. *p* < 0.05 (Tukey HSD test)

The results showed that the application of 0.1 and 10 μM IAA to roots of Fe-sufficient plants caused a dose-dependent effect on both PS root release and root *TaNAAT* gene expression, compared with non-treated Fe-sufficient plants (Fig. 6a, b). Thus, the lowest dose of IAA caused a prompt (4 h) but transient increase in PS root release (Fig. 6a), which was associated with a slight but significant increase in root *TaNAAT* expression at 4 h, while the highest dose caused an increase of both PS root release and *TaNAAT* expression in roots with time (Fig. 6b).

### Fe-deficiency was not associated with significant increases in IAA root and shoot concentrations

In order to investigate whether the above described effects of IAA on the regulation of Fe-deficiency root responses were accompanied by an increase in the IAA root and shoot concentrations, we analyzed the IAA concentration in both roots and shoots of Fe-sufficient plants, as well as in Fe-deficient plants before and after receiving Fe-foliar resupply.

**Fig. 7:**
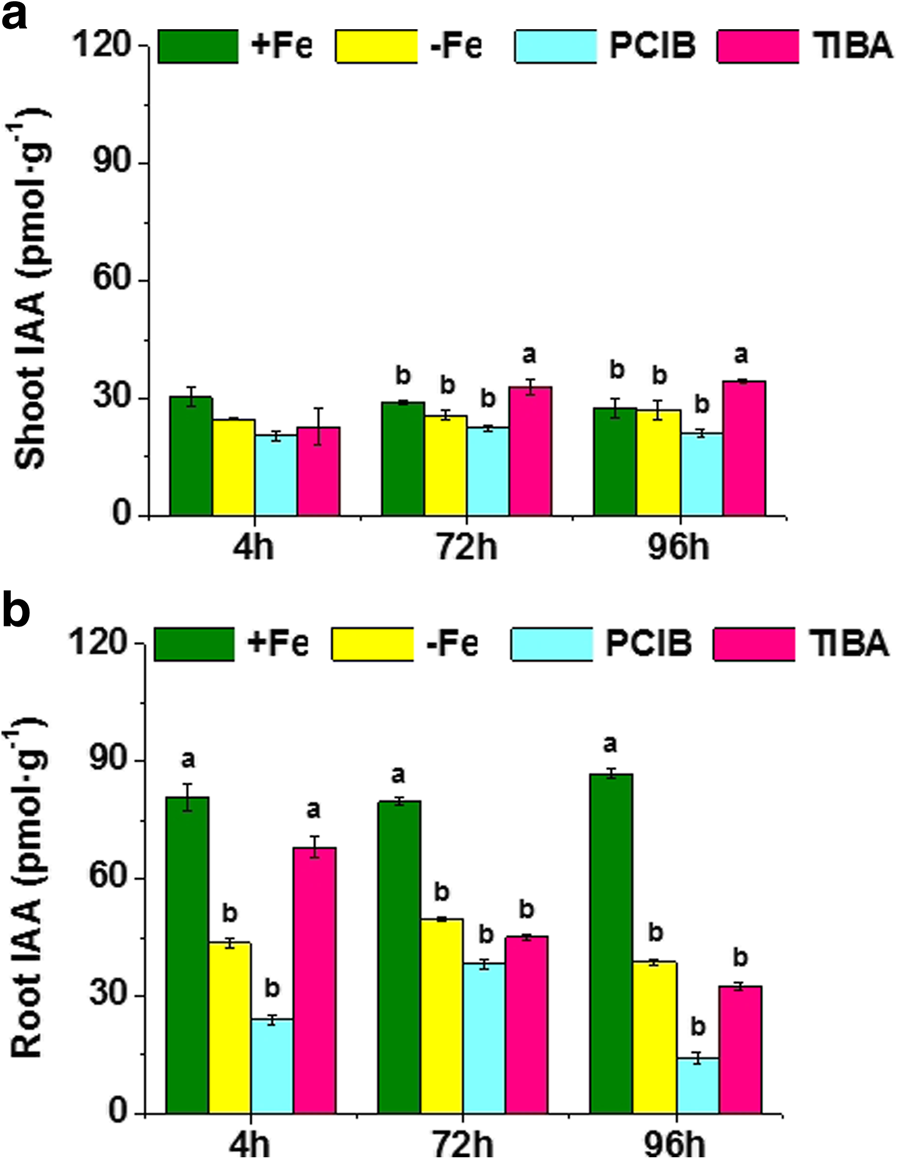
Plant concentration of Indole Acetic Acid (IAA) on wheat plants under Fe deficiency and treated with IAA inhibitors. (**a**) IAA concentration in shoots of Fe-sufficient (+Fe), Fe-starved (-Fe), and Fe-starved plants treated with PCIB (200 μM) or TIBA (50 μM); in pmol per g of root fresh weight. Harvests were performed at 4, 72 and 96 h of treatment, which corresponded to the three columns per treatment. Average of 3 replicates with 20 plants each. *p <* 0.05 (Tukey LSD test). **b** IAA concentration in roots of Fe-sufficient (+Fe), Fe-starved (-Fe), and Fe-starved plants treated with PCIB (200 μM) or TIBA (50 μM); in pmol per g of root fresh weight. Harvests were performed at 4, 72 and 96 h of treatment, which corresponded to the three columns per treatment. Average of 3 replicates with 20 plants each. *p* < 0.05 (Tukey HSD test)

**Fig. 8:**
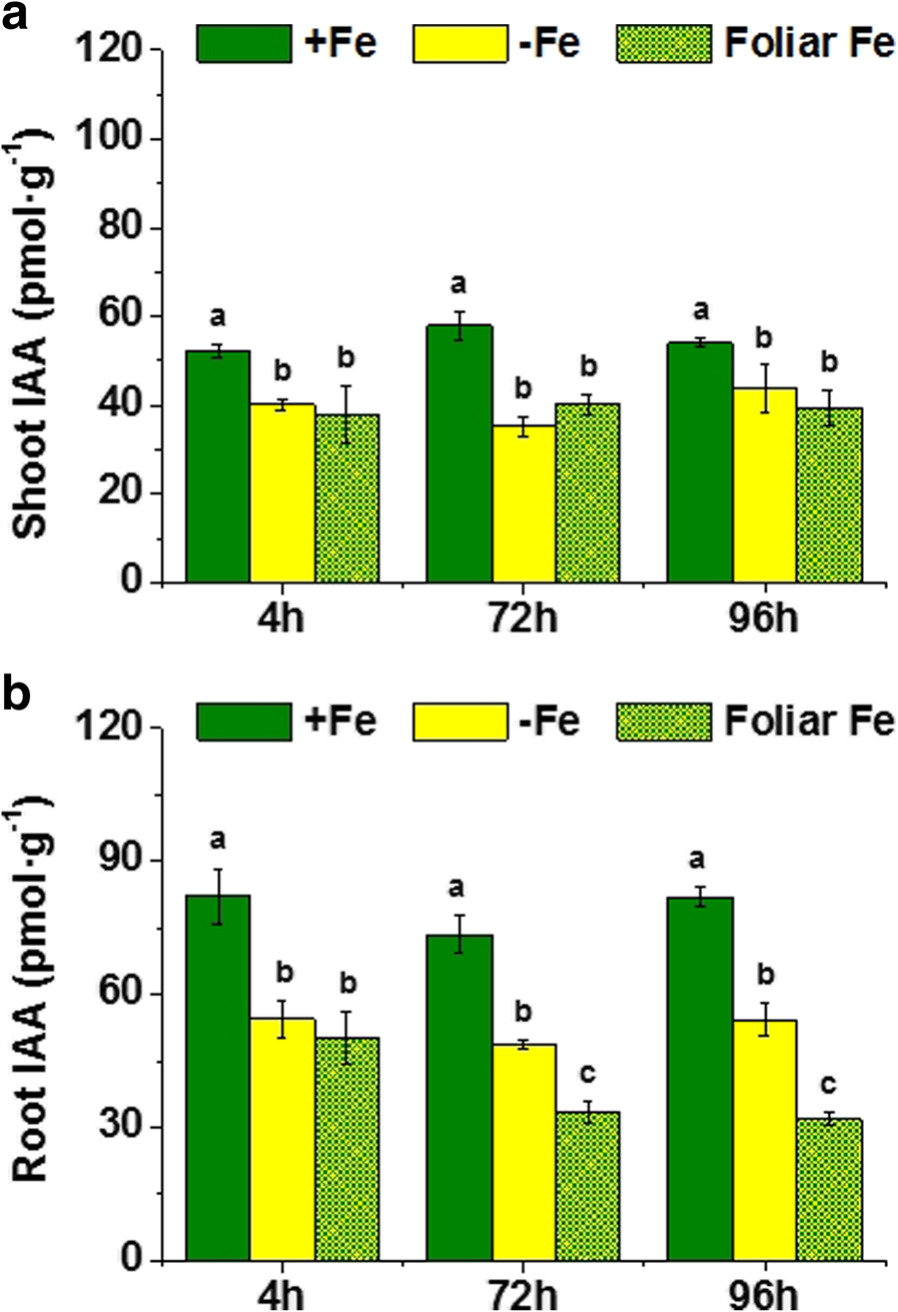
Plant concentration of Indole Acetic Acid (IAA) on wheat plants under Fe deficiency with Fe ressuply. (**a**) IAA concentration in roots of Fe-sufficient (+Fe), Fe-starved (-Fe), and Fe-starved plants supplied with Fe applied on leaves in pmol per g of root fresh weight. Harvests were performed at 4, 72 and 96 h of treatment, which corresponded to the three columns per treatment. Average of 3 replicates with 20 plants each. *p <* 0.05 (Tukey LSD test). **b** IAA content in roots of Fe-sufficient (+Fe), Fe-starved (-Fe), and Fe-starved plants supplied with Fe applied on leaves in pmol per g of root fresh weight. Harvests were performed at 4, 72 and 96 h of treatment, which corresponded to the three columns per treatment. Average of 3 replicates with 20 plants each. *p* < 0.05 (Tukey HSD test)

The results showed that plants subjected to Fe deficiency instead of presenting an increase of IAA root concentration, showed a significant decrease (Fig. 7a). No differences were observed between the shoot IAA concentration of Fe-sufficient and deficient plants (Fig. 7a), while a significant decrease was observed in the root of Fe-deficient plants treated with TIBA and PCIB (Fig. 8).

Interestingly, Fe-foliar resupply to Fe-deficient plants reduced IAA root concentration even more than Fe deficiency alone, over time (Fig. 7b).

## Discussion

Whereas the molecular network involved in Strategy I and Strategy II plant root responses to Fe-deficiency have been well established [4, 8, 9], the signaling pathways governing these processes at the whole plant level are less well known, especially in the case of Strategy II plants. In Strategy I plants, a number of studies have shown that, though roots are able “per se” to activate Fe-starvation root responses, the systemic regulation of these responses depends on the Fe-status in the shoot [14–16, 19]. Several studies have proposed that the dominant role of the shoot in the activation of Fe-deficiency responses in roots is probably also present in Strategy II plants [8]. However, there is no experimental evidence supporting this assessment. In Strategy I plants, this question has been tackled by using split-root experiments and/or Fe-foliar application [14–16, 19]. While Fe-foliar application allows us to study whether Fe-deficiency root responses are governed by shoot Fe-status (systemic regulation), split-root experiments also allow the discrimination between local and systemic shoot-root signal regulation [14, 16, 19, 51]. In the present study, we have employed the Fe-foliar re-supply approach in order to investigate the role of the shoot in the fine control of the activation of the studied Fe-deficiency root responses in wheat. If Fe-deficiency in the shoot governs the activation of these Fe-deficiency responses in roots, the application of available Fe to leaves of plants suffering Fe-deficiency must significantly reduce the expression of Fe-deficient root responses, as observed in Strategy I plants. Our results clearly showed this fact. The Fe foliar application to Fe-starved and deficient plants led to the gradual reduction in both PS root release and *TaNAAT* gene expression compared to control Fe-deficient plants. On the other hand, previous studies have shown that other genes concerning DMA synthesis (i.e. *TaDMAS1*) are upregulated in wheat root under Fe deficiency but they remain unchanged in the shoot [52]. These results indicate that the presence of Fe-deficiency in leaves plays a major role in the activation of the studied Fe-starvation root responses in wheat, a Strategy II plant species.

With regard to the nature of the signaling pathways involved in the activation of Fe-deficient root responses in graminaceous plants, current knowledge is very limited. In Strategy I plants a number of studies have shown that this process is rather complex and possibly involves both repressive and activating signals likely related to Fe-transporter compounds along with hormone-regulated signaling pathways [7, 8, 12, 17, 18, 25]. Thus, hormonal pathways integrate a coordinated action of auxin in shoot and root [28–31] and ethylene-NO in roots [17–19]. In graminaceous plants, recent results showed that many genes related to plant hormone signaling (ethylene, auxin, nitric oxide, cytokinin, jasmonate…), responded to Fe-deficiency stress, thus suggesting the involvement of a complex hormonal signaling network in the fine regulation of Fe-deficiency induced root responses [48, 53].

Some results have suggested that ethylene might play a role in the positive regulation of Fe-deficiency root responses [38]. If this is correct, the application of inhibitors of ethylene functionality must block the expression of Fe-deficiency root responses in Fe-starved and deficient graminaceous plants. However, our results clearly showed that the application of both Co^2+^, an inhibitor of ethylene synthesis, and STS, an inhibitor of ethylene action, did not cause any decrease in the expression or intensity of PS root release and *TaNAAT* gene expression compared with Fe-deficient plants in wheat. In principle, this fact indicates that ethylene does not positively regulate the evolvement of these Fe-deficiency root responses in wheat. This conclusion is in line with the results reported by Romera et al. [37], who did not observe any increase in ethylene root production in barley, maize and wheat plants subjected to Fe-deficiency, and Welch et al. [36] who did not observe any change in PS root release upon ACC (a precursor of ethylene synthesis) application to barley plants. The results reported by Wu et al. [38], who observed an increase in ethylene root production in Fe-deficient rice plants, might be due to the fact that rice shares some Strategy I and Strategy II Fe-deficiency root responses [8].

On the contrary, the application of STS caused a slight but significant increase in PS root release after 4 h, although it did not affect the root *TaNAAT* up-regulation associated with Fe-deficiency. These results are in line with those reported by Welch et al. [36], who observed an increase of PS root efflux upon AOA application in barley. In principle, these results may be linked to the fact that ethylene competes with nicotinamine (NA) for the S-Adenosyl methionine availability for their respective synthetic pathways, and blocking ethylene function may favor NA-pathway and PS biosynthesis under Fe starvation. However, the physiological meaning of this result is dubious, since ethylene function is crucial for normal plant development and blocking ethylene functionality negatively affect major metabolic pathways and physiological processes. In fact, Welch et al. [36] reported that despite the increase in PS root efflux caused by AOA, both plant growth and Fe root uptake were clearly impaired by AOA application.

Several studies have clearly stated that NO was able to improve internal Fe availability in maize [32]. This fact might be related in some way to the NO-ability to promote ferritin accumulation and Fe-storage in leaves [33]. In this framework, it would be possible that NO also plays a role in the regulation of Fe-deficiency root responses in graminaceous plants through the control of Fe remobilization within the plant. In this sense, it was is possible that the inhibition of NO signaling pathway by c-PTIO would decrease ferritin and increase free Fe in the shoot thus alleviating Fe deficiency and decreasing PS root release and root TaNAAT up-regulation. Nevertheless, our results showed that NO does not affect the PS root release associated with Fe deficiency. Similar results were observed concerning root *TaNAAT* gene expression, which presented up-regulation levels similar to those of Fe-deficient plants. As in the case of STS root application, c-PTIO caused a slight but significant increase of PS root release compared with Fe-deficient plants after 4 h. In contrast to the dubious physiological meaning of effects derived from the inhibition of ethylene action, the effects on Fe-deficiency root responses resulting from the NO-action inhibition might be much more meaningful. In this sense, as stated above, NO-mediated improvement of internal Fe availability might be linked to its ability to modulate ferritin accumulation in leaves [32, 33, 49]. Ferritins are proteins located in the chloroplasts where accumulation is induced by an excess of iron. NO mediates transcriptional regulation of ferritin genes by regulating an iron-dependent regulatory sequence (IDRS), a cis-acting element that depresses the expression of several ferritin genes under Fe overload [33]. In this context, blocking NO functionality may also affect ferritin biosynthesis in leaves of Fe-deficient plants, thus affecting ferritin gene *TaFer1* expression. However, c-PTIO application did not increase the down-regulation of *TaFer1* associated with Fe-deficiency. This result do not exclude that *TaFer1* down-regulation is not linked to the inhibition of NO-dependent signaling pathways but that other signals trigger by Fe deficiency, which may be related to other IDRS elements [54], are also involved.

The relevant role played by auxin in the activation of Fe-deficiency root responses in Strategy I plants has been well established [28–31]. However, auxin action in the activation of Strategy II plant responses has not been directly studied yet. Some studies reported that genes closely related to auxin transport [44] and function [43] seem to be involved in the regulation of Fe-homeostasis in rice. These results suggested that auxin could also play some role in the activation of Fe-deficiency root responses in graminaceous plants. However, the fact that rice also has Strategy I Fe-deficiency root responses [8] makes it difficult to investigate this issue using this plant species. Our results show that the application of PCIB, an inhibitor of IAA-action, caused a gradual decrease of both PS root release and root *TaNAAT* gene expression over time in comparison with Fe-deficient plants. This result indicates that IAA is essential for the activation of Fe-deficiency root responses in wheat.

The results obtained from TIBA application, an inhibitor of IAA-transport, were also meaningful. TIBA-treated plants showed a rapid decrease in the *TaNAAT* gene up-regulation caused by Fe-deficiency that was expressed 4 h upon TIBA application. However this effect disappeared after longer times. On the other hand PS root release had a different pattern of variation after TIBA application, showing no increase for all harvest times. In principle, these results suggest that auxin controls *TaNAAT* expression and PS root release through different pathways. The fact that PCIB blocked both *TaNAAT* expression and PS root release suggests that the effects observed with TIBA may be related to the IAA distribution in the different root zones where the regulated biochemical events occur. Dependence of root local processes on IAA local root distribution has been previously reported in Arabidopsis concerning IAA-mediated root morphological changes induced by Fe-deficiency [55–57]. Further studies are needed in order to clarify this issue.

In contrast to Strategy I plants [29], Fe-deficiency did not cause any increase in the root concentration of IAA, but a significant decrease in both root and shoot. Furthermore, the application of Fe on the leaves of Fe-deficient plants caused an additional decrease of IAA root concentrations. These results suggest that IAA local distribution in roots might be much more relevant than total plant IAA concentration in the main role of IAA in the activation of Fe-deficiency root responses in graminaceous plants. In this sense, other authors also observed that some auxin-dependent processes, such as the alteration in the pattern of lateral root (LR) formation and emergence in response to phosphate (Pi) availability in Arabidopsis, were related to changes in auxin receptor sensitivity but not to root IAA concentration [58]. Nevertheless, the conversion of IAA into conjugated forms with higher auxin activity is also possible [59]. In fact, the decrease in IAA observed in our experiments in plants subjected to Fe starvation was associated with a concomitant increase in several IAA conjugated forms, principally IAA-aspartate and IAA-Glutamate (unpublished results). Although aminoacid conjugation has been related to hormonal activation in the case of JA and JA-isoleucine [60] and with hormonal deactivation in the case of IAA [61], recent studies showed that IAA-Aspartate has a regulatory function in pathogen infection and plant defense [59]. It is therefore possible that any of the conjugated forms promoted by Fe deficiency is involved in root plant responses to Fe deficiency in graminaceous. More studies are required in order to shed light on this issue.

Our results suggest that auxin might also be involved in the shoot to root signaling network regulating root responses to Fe deficiency in Strategy II plants. On the one hand, as discussed earlier, both TIBA and PCIB root applications to Fe-deficient plants were associated with a decrease in IAA root concentration, with these effects being linked to significant decreases in PS root. On the other hand, IAA application was able to promote both root *TaNAAT* up-regulation and PS root release in Fe-sufficient wheat plants. However, the time-course pattern of the variation of the two evaluated parameters in Fe-sufficient plants upon IAA application was different from that of Fe-deficient plants. This fact indicates that other factors, which may be related to a kind of repressive signal as in the case of Strategy I system [19], are probably involved in the complex signaling network responsible for the shoot-mediated regulation of Fe-deficiency root responses in graminaceous plants.

## Conclusions

**Fig. 9:**
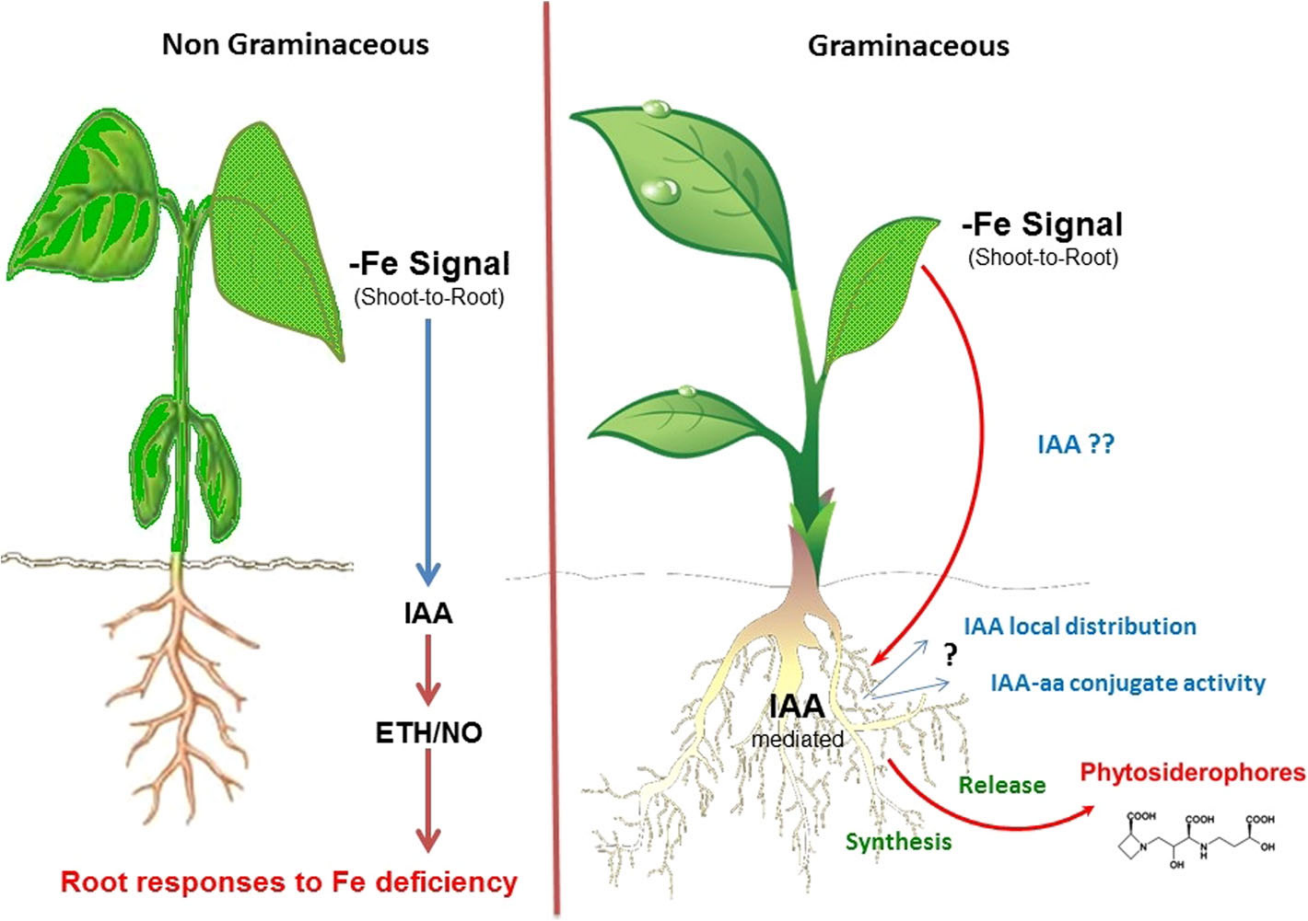
Proposed mechanism for the role of indole acetic acid (IAA) in the modulation of root responses to Fe deficiency in non graminaceous and graminaceous plants (ETH: ethylene; NO: nitric oxide) (This figure was drawn by M.G, R.B. and J.M. G-M)

The results obtained in the present study show that the evolvement of Fe-deficiency in the shoot regulates the activation of the studied Fe-deficiency root responses. Furthermore, auxin plays an essential role in the control of the release of PS from the root to the rhizosphere in wheat plants subjected to Fe starvation and deficiency. The results were also compatible with a role of IAA in the shoot regulation of Fe-deficiency root responses in Fe-deficient wheat plants (Fig. 9). The results suggest the possible relevance of IAA local concentration and redistribution in specific root regions as well as the involvement of IAA conjugated forms with higher auxin activity, in the whole action of IAA on the activation of Fe-deficiency root responses. On the other hand, ethylene and NO did not clearly modulate the studied Fe-deficiency root responses in wheat. However, an indirect effect of NO cannot be ruled out since this molecule is able to improve internal Fe availability and use in the shoot, thus delaying Fe-deficiency response.

## Abbreviations

ABCB14: Gene involved in both auxin transport and Fe homeostasis
ACC: 1-aminocyclopropane-1 carboxylic acid
ANOVA: Analysis of variance
AOA: Amino-oxiacetic acid
ARF12: Transcription factor that activates the auxin response gene
BRS: Brassinosteroids
c-PTIO: 2-phenyl- 4,4,5,5-tetramethylimidazoline-3-oxide-1-oxyl
DMSO: Dimethyl sulfoxide
ETH: Ethylene
FER: Ferritin
H^+^-ATPase: proton-pumping ATPase
HvNAAT-A/B, HvNAS, IDS3: barley genome fragments
IAA: Indolacetic acid
ICP-OES: Inductively coupled plasma optical emission spectrometry
IDRS: Iron-dependent regulatory sequence
MAs: Mugineic acids
NAAT: Nicotianamine aminotransferase
NAS: Nicotianamine synthase
NO: Nitric oxide
PCIB: 2-(p-chlorophenoxy)-2-methylpropionic acid
PS: Phytosiderephores
SAM: S-adenosyl-L-methionine
STS: Silver thiosulphate
TIBA: 2,3,5-triiodobenzoic acid
TOM1, YSL15 & DMAS1: genes involved in Strategy II responses under Fe deficiency
